# *Nigrospora oryzae* Causing Leaf Spot Disease on *Chrysanthemum* × *morifolium* Ramat and Screening of Its Potential Antagonistic Bacteria

**DOI:** 10.3390/microorganisms11092224

**Published:** 2023-09-01

**Authors:** Haodong Sha, Xinyi Liu, Xiaoe Xiao, Han Zhang, Xueting Gu, Weiliang Chen, Bizeng Mao

**Affiliations:** 1 Institute of Biotechnology, College of Agriculture & Biotechnology, Zhejiang University, Hangzhou 310058, China; 2 Ministry of Agriculture Key Lab of Molecular Biology of Crop Pathogens and Insects, Hangzhou 310058, China; 3 Zhejiang Tongxiang Hangbaiju Technology Academy, Tongxiang 314500, China

**Keywords:** *Nigrospora oryzae*, leaf spot disease, *Chrysanthemum morifolium*, *Bacillus siamensis*, antagonisms

## Abstract

*Chrysanthemum* × *morifolium* Ramat. is a famous perennial herb with medicinal, edible, and ornamental purposes, but the occurrence of plant diseases can reduce its value. A serious disease that caused leaf spots in *C. morifolium* appeared in 2022 in Tongxiang City, Zhejiang Province, China. The *C. morifolium* leaves with brown spots were collected and used for pathogen isolation. By completing Koch’s postulates, it was proven that the isolate had pathogenicity to infect *C. morifolium*. It was determined that the pathogen isolated from chrysanthemum leaves was *Nigrospora oryzae*, through morphology and a multilocus sequence analysis method using a combination of the internal transcribed spacer gene (ITS), beta-tubulin gene (TUB2), and translation elongation factor 1-alpha gene (TEF1-α). This is the first report of *C. morifolium* disease caused by *N. oryzae* in the world. Through dual culture assay on PDA plates, 12 strains of bacteria with antagonistic effects were selected from 231 strains from the *C. morifolium* phyllosphere, among which *Bacillus siamensis* D65 had the best inhibitory effect on *N. oryzae* growth. In addition, the components of a strain D65 fermentation broth were profiled by SPME-GC-Q-TOF analysis, providing a foundation for further application and research of biological control.

## 1. Introduction

*Chrysanthemum* × *morifolium* Ramat., a perennial herb native to China, is widely distributed in China and has a long history of cultivation [1]. Since ancient times, Chinese people have been fond of using chrysanthemums as a cultural carrier; there exist many poems praising the chrysanthemum and endowing it with a noble character and the quality of indifference to fame and wealth. The chrysanthemum is also an important traditional Chinese medicine [2]; it contains volatile oils, triterpenes, flavonoids, phenolic acids, and other phytometabolites, which have antimicrobial, antiviral, antioxidant, antiaging, anticancer, and anti-inflammatory activities. It is popular as well as important, and is usually used as food and tea in China [3]. With the development of the times and cultural exchanges, the chrysanthemum has spread all over the world. Now, it has a high economic value ranking second in the cut flower trade after the rose [4].

During plant growth, fungal diseases often affect plant development. According to reports, over 19,000 fungi are known to cause diseases in crop plants worldwide [5]. The leaf is an essential organ of most plants that collects and converts solar energy for plant growth [6]. The phyllosphere, including leaves, has been estimated to make up around 60% of the biomass across all taxa on the earth, making it a key habitat for microbial organisms [7]. Pathogenic fungi, as a component of phyllosphere microbial organisms, often induce plant leaf diseases [8]. On the chrysanthemum, pathogenic fungi—including the genera *Ascochyta*, *Septoria*, and *Verticillium*—often cause damage to its leaves [9].

There exist a huge diversity of microbial communities in the phyllosphere of plants. In addition to pathogenic fungi, there are also other microorganisms distributed in the phyllosphere that are much more diverse and numerous than pathogenic fungi [10,11]. These microbial communities are collectively referred to as the phyllosphere microbiota [12]. Since these microorganisms occupy ecological niches that are the same as, or similar to, those of pathogenic fungi, there is a large number of antagonistic and competitive relationships between these microbial populations. These microorganisms with antagonistic effects can be screened out as Biocontrol Agents (BCA) which, when applied as a biological control, can reduce the use of pesticides [13].

There are many varieties of *C. morifolium* with different attributes [1,3]. For chrysanthemums used for ornamental purposes, the occurrence of leaf diseases reduces the overall aesthetic feeling of chrysanthemums and damages their ornamental value [14]. For edible and medicinal chrysanthemum varieties, leaf diseases reduce the yield of chrysanthemums by affecting photosynthesis and plant health [15,16]. Therefore, a study about chrysanthemum leaf disease is of great significance.

Tongxiang City of Zhejiang Province is a famous chrysanthemum production area on a nationwide scale, which contributes 50% of the yields of edible chrysanthemum in China [16]. In October 2022, a chrysanthemum leaf disease occurred widely in Tongxiang, where the disease incidence was estimated to be more than 10%.

In order to identify the pathogen that caused the disease and obtain potential antagonistic bacteria for the prevention and control of the disease, we conducted the following work in this study: (1) isolation and identification of the pathogen causing *C. morifolium* leaf spot disease; and (2) isolation of the phyllosphere bacteria of the chrysanthemum and the screening out of bacteria that have antagonistic effects on the pathogen. This work is of great significance for the research and biological control of leaf diseases in *C. morifolium*.

## 2. Materials and Methods

### 2.1. Pathogen Isolation and Culture Conditions

The symptomatic leaves of chrysanthemum were collected from Shimeng Town, Tongxiang City, Zhejiang Province, China (120.46° E, 30.62° N; altitude: 5 m). The collected samples were taken to the laboratory and immediately processed. Strains were isolated according to Fang’s method with slight modification [17]. Briefly, a small section (about 5 × 5 mm) taken at the border between the infected and healthy parts of the disease leaf was aseptically cut and surface-sterilized in 75% alcohol for 30 s, washed with sterile water 2 times followed by 3% sodium hypochlorite solution for 1 min, and rinsed 3 times in sterile water. Leaf sections were dried on sterilized filter paper and plated on potato dextrose agar (PDA) medium containing 50 μg/mL streptomycin and 50 μg/mL penicillin. The plates were incubated under 25 °C in the dark for 4 days. Pure mycelia were obtained by hyphae tip separation; the mycelium on the outside of the colony was picked with a sterilized toothpick and placed on new plates for a new round of culture until the pure colony was obtained.

### 2.2. Pathogenicity Tests and Morphological Observations

In order to verify the pathogenicity of the isolated fungi, according to Zhang’s method with small modification [18], the leaves and branches were put on a moist sterile filter paper in Petri dishes, a 7 × 7 mm mycelium plug was placed on the wounded leaves, and the sterile PDA was placed on the wounded leaves as the controls. The dishes were incubated in a growth chamber under artificial light (12/12 h light/dark) at 30 °C and 80% humidity, and the symptoms were observed after 5–7 days. The microstructures of spores and hyphae were observed with a Nikon Eclipse Ni microscope equipped with a Nikon DS-Fi2 digital camera after cultivation for 2 weeks.

### 2.3. Phylogenetic Analysis

#### 2.3.1. Genomic DNA Extraction and PCR Amplification

The mycelia cultured on PDA for 7 days was scraped off with a sterile scalpel, frozen with liquid nitrogen, and then transferred to a mortar for grinding. The genomic DNA of the isolates was extracted by using the Plant Genomic DNA kit (Tiangen, China). The primer pair ITS1/ITS4 was used to amplify the internal transcribed spacer region (ITS) [19], Bt2a/Bt2b was used to amplify the beta-tubulin gene (TUB2) [20], and TEF1-728F/EF2 was used to amplify the translation elongation factor 1-alpha gene (TEF1-α) [21,22]. The primers are summarized in Table 1. All amplification reactions were performed in a total volume of 20 μL mixture consisting of 10 μL of 2 × Phanta Flash Master Mix (Vazyme, Nanjing, China), 7 μL ddH_2_O, 1 μL of each forward and reverse primer, and 1 μL of DNA template (1 ng/μL). The amplification conditions were as follows: initial denaturation at 98 °C for 30 s, followed by 35 cycles of denaturation at 98 °C for 10 s, annealing at 55 °C for 5 s, and extension at 72 °C for 10 s, with a final extension step at 72 °C for 5 min. The obtained sequences were sent to Zhejiang Youkang Biotechnology Co., Ltd. (Hangzhou, China) for sequencing.

#### 2.3.2. Phylogenetic Analysis

Phylosuite software v1.2.3 was used as a platform for phylogenetic analysis [23]. The ITS, TUB2 and TEF1-α sequences were aligned using MAFFT v7.505 separately using the “--auto” strategy and normal alignment mode [24]. After concatenation, the best partitioning scheme and evolutionary models for 3 pre-defined partitions were selected using PartitionFinder2 v2.1.1, with all algorithms and the AICc criterion [25]. Then Bayesian Inference phylogenies were inferred using MrBayes v3.2.7a under the partition model (2 parallel runs, 2,000,000 generations), in which the initial 25% of sampled data were discarded as burn-in [26]. Figtree software v1.4.4 (http://tree.bio.ed.ac.uk/software/figtree/ (accessed on 25 July 2023)) was used to display the tree. GenBank accession numbers obtained in this study are listed in Supplementary Table S1, and several reference sequences of the *Nigrospora* genus from GenBank (http://www.ncbi.nlm.nih.gov (accessed on 24 July 2023)) used in the phylogenetic analysis are listed in Supplementary Table S2.

### 2.4. Isolation of Bacteria from the Phyllosphere

In order to obtain culturable microbial isolates from the chrysanthemum phyllosphere, Zhan’s method was used with some modifications [27]. Firstly, 5 g of healthy chrysanthemum leaves were collected from the location where the disease occurred and those chrysanthemum leaves were disinfected with 75% ethanol for 60 s. Then, they were washed three times with sterile water and dried on sterilized filter paper. The treated leaves were placed in a mortar, 5 mL of phosphate buffer saline was added for grinding, and the grinding fluid was diluted 2, 5, and 10 times. A quantity of 50 μL of the above grinding liquid was dropped into the center of a Luria–Bertani (LB) medium plate and evenly coated. It was then incubated under dark conditions at 30 °C for 3 days. Afterwards, single colonies were picked using sterilized toothpicks and inoculated onto a new culture medium using the streak plate method, each cultured as an isolate of phyllosphere bacteria.

### 2.5. Screening and Identification of Antagonistic Bacteria

Fungi isolated from diseased chrysanthemum leaves and phyllosphere bacteria isolated from healthy chrysanthemum leaves were used for this experiment. Firstly, the initial screening was carried out. After 5 days’ cultivation of fungi on PDA, a small portion of the mycelium was picked using sterilized toothpicks and placed on one side of a new PDA plate, while different strains isolated from the chrysanthemum leaf space were placed on the other side. Subsequently, the plate was incubated under dark conditions at 27 °C for 5 days to observe whether the growth of fungi was inhibited. Then, the plates were screened and the strains that inhibited fungal growth were preserved.

In order to identify the strains which had antagonistic ability, 27F/1492R primer was used to amplify 16 s rRNA gene sequences (Table 1) [28]. The gene extraction, PCR reaction system, and procedure were the same as described in Section 2.3.1. Obtained sequences were blasted with type materials available at NCBI (http://www.ncbi.nlm.nih.gov (accessed on 11 August 2023)) to obtain the highest hit for taxon and strain. The EasyID Microbial Biochemical Reagent Kit (Guangdong Huankai Biotechnology Co., Ltd., Guangzhou, China) was used for testing the mannitol fermentation, glucose utilization under anaerobic conditions, amylolysis, nitrate reduction abilities, and V-P of each strain.

### 2.6. In Vitro Antagonism against N. oryzae

The antagonism ability of each antagonistic strain screened from the previous study was determined by dual culture assay on PDA plates [29]. In general, we took a 6 mm agar–mycelium plug obtained from *N. oryzae* that had been cultured on PDA medium for 5 days, placed in the center of a new PDA plate, and incubated in the dark conditions of 27 °C for 1 day. Then we added 0.5 μL of the antagonistic suspension (1 × 10^6^ CFU/mL) dropwise to 4 areas of the PDA plate, each positioned 1 cm from the edge of the plate. PDA with dropwise water was used as a control. The plates were then cultured at 27 °C for 6 days in the dark, and the colony radius of *N. oryze* was measured in mm using a vernier caliper, retaining two decimal places. The experiment was repeated twice in three replicates. GraphPad Prime v8.0 was used to calculate the mean and quartiles of the colony radius. One-way ANOVA was used to obtain the difference between each strain. The statistical results are displayed as a box plot.

### 2.7. Identification of the Active Metabolites from the Antagonistic Strain

The strain with the best antagonistic ability was used to determine the chemical composition of its fermentation broth. After 48 h of cultivation, the strain was inoculated with sterile toothpicks into a 400 mL conical flask containing 250 mL LB medium, and incubated under dark conditions at 25 °C at 200 r/min for 3 days. The fermentation broth was obtained by centrifuging at 10,000 rpm for 10 min. LB medium was used as the control.

SPME-GC-Q-TOF (Agilent 7250 GC/Q-TOF, USA) was used to determine the composition of the antagonistic bacterial fermentation broth. Samples from the PAL Sampler were extracted for 15 min at 80 °C, incubated for 20 min, then heated and stirred for 80 min at 200 rpm. Then the sample was pre-desorbed for 2 min at 240 °C, post-desorbed for 2 min, and then pre-washed and post-washed for 35 min. After the test, the results were compared with the National Institute of Standards and Technology (NIST) library to obtain the possible composition of compounds in the antagonistic bacterial broth.

We discarded compounds with a match factor below 90, and then rounded out compounds containing silicon to ensure the accuracy of the test results. We compared the results of the fermentation broth with LB medium, and obtained compounds that were present in the fermentation broth but were not present in LB medium, which are components that may have the ability of inhibit growth of pathogenic fungi in the fermentation broth.

## 3. Results

### 3.1. Plant Disease Symptoms and Pathogenicity Test

**Figure 1:**
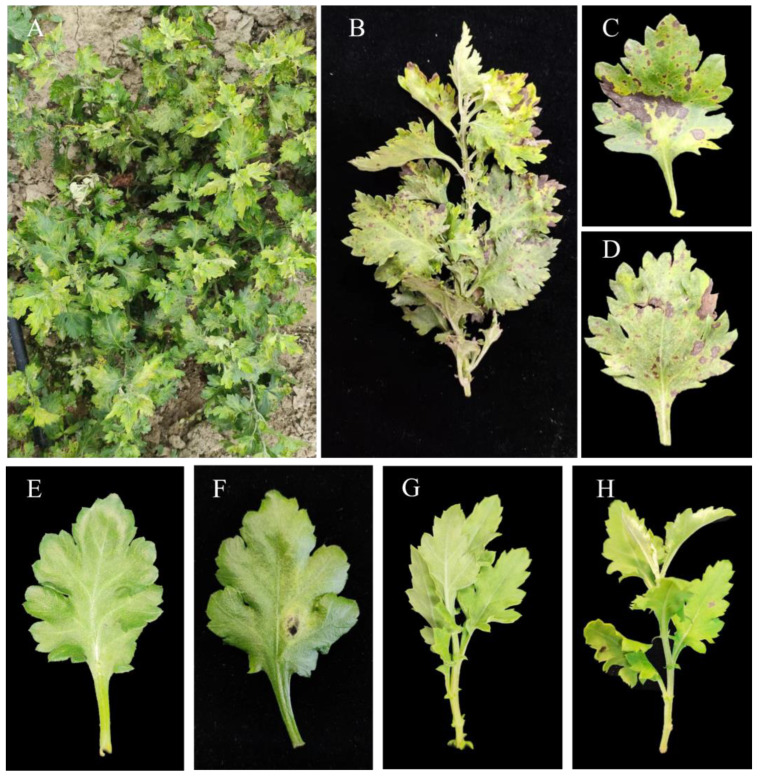
Symptoms of disease in *C. morifolium* and pathogenicity test. (**A**) Field symptoms. (**B**) Diseased branches near the top of the chrysanthemum. (**C**,**D**) Symptoms of leaf spot disease. (**E**) Leaf 5 days after inoculation with sterile PDA plug. (**F**) Leaf 5 days after artificial inoculation. (**G**) Branch before inoculation. (**H**) Branch 7 days after inoculation.

The disease occurs widely on chrysanthemum leaves. The typical disease symptoms are shown in (Figure 1A–D). The disease spot is brown to dark brown, and the shape of the disease spots is an irregular small dot. In severe cases, each small spot can combine to form a large irregular spot. When the disease occurs seriously, the entire shoot and leaf are infected. For a single plant, the disease occurs from the top new leaf to the bottom old leaf, but the top new leaf is affected more seriously. As for a single leaf, the number and severity of the disease spots at the edge of the leaf are greater than those at the center of the leaf.

For the pathogenicity test, dark brown spot symptoms were observed on the inoculation site 5 to 7 days after being inoculated (Figure 1F,H). The symptoms were consistent with the disease observed in the field, while no obvious symptoms were observed in the control group (Figure 1D). In addition, the fungal isolates were re-isolated from infected leaves, which fulfilled Koch’s postulates.

### 3.2. Morphological Characterization

**Figure 2:**
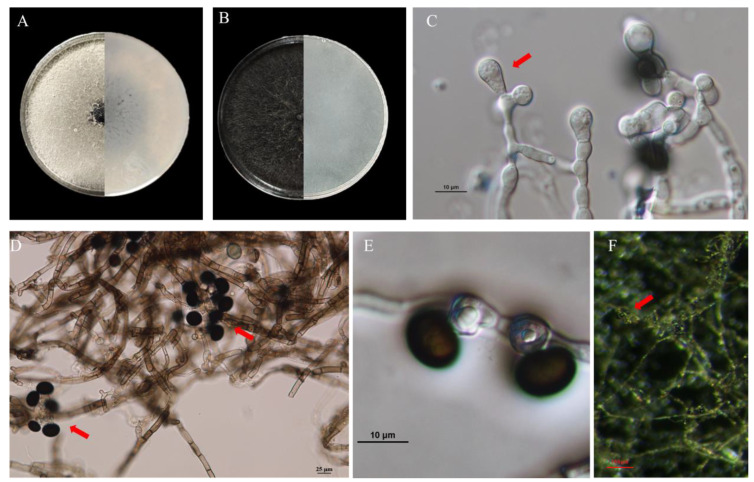
Morphological characterization. (**A**) Colony on PDA medium after 7 days. (**B**) Colony on PDA medium after 21 days. (**C**) Ongoing conidia. (**D**,**E**) Conidia and hyphae observed with optical microscope (**F**) Conidia and hyphae observed with stereomicroscope. The red arrow points to conidia.

The isolate was cultured on PDA, and the colony of the isolate was white to grayish white, then gradually turned black from the center of the colony with the production of spores, until it turned completely black during 3 weeks of incubation (Figure 2A,B).

The hyphae were branched and septate with a transparent or translucent black color, the conidia were transparent initially at the top or middle of the hypha, and then turned into opaque black gradually when mature (Figure 2C–F). They were one-celled, spherical or near-spherical with a smooth surface, and the sizes of the spores were 11.2–15.97 × 10.64–15.91 μm (av. = 13.95 ± 1.20 × 13.40 ± 1.48 μm, n = 25). These characteristics of the pathogen were similar to the descriptions of *Nigrospora oryzae* [30,31].

### 3.3. Molecular Characterization and Phylogenetic Analysis

Ten isolates with similar morphological characteristics were isolated from the leaves of chrysanthemum. To further confirm the identity of the pathogen, three isolates, TaxaJA, TaxaJE, and TaxaJF, were selected to construct the phylogenetic tree using the ITS, TUB2, and TEF1-α genes. The final concatenated DNA sequences of each isolate had 1450 bp including ITS (505 bp), TEF1-α (526 bp), and TUB2 (419 bp). *Arthrinium malaysianum* served as an outgroup. The topology of the phylogenetic tree was consistent with Li’s research [32].

**Figure 3:**
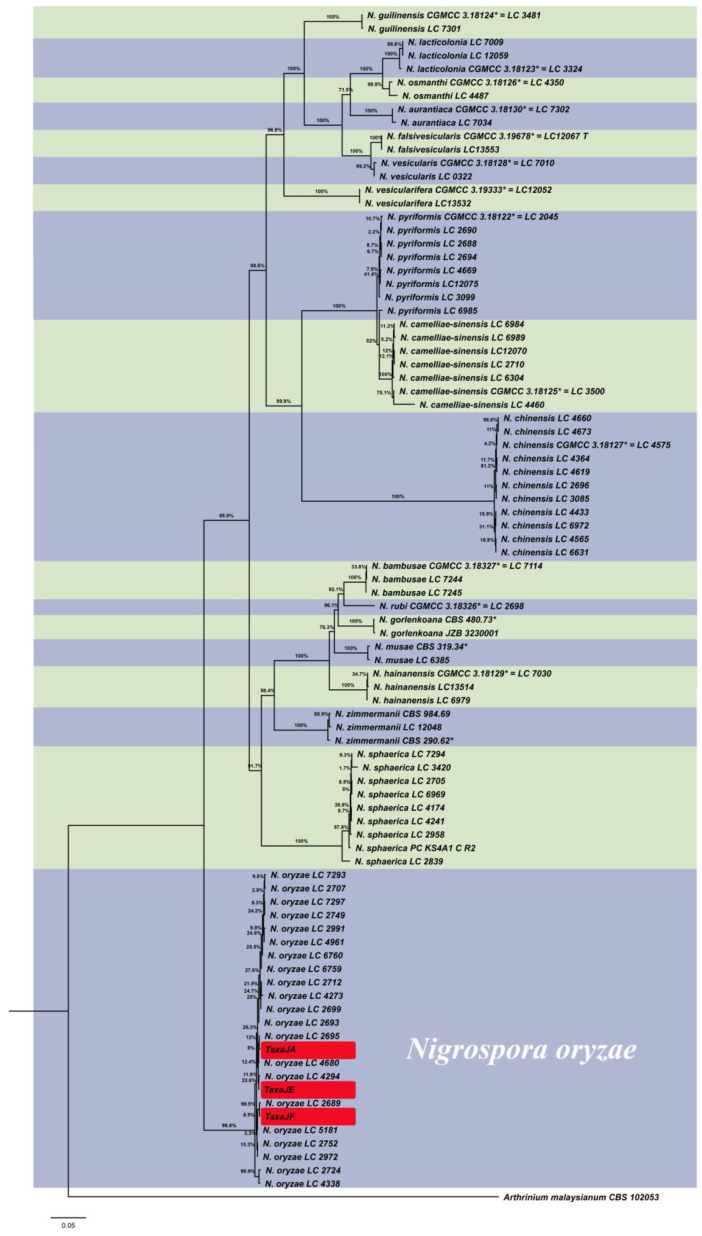
Bayesian inference was used to construct the phylogenetic tree using ITS, TUB2, and TEF1-α sequences of *Nigrospora* species. *Arthrinium malaysianum* served as outgroup. The asterisk represents the type materials.

According to the phylogenetic tree, all three isolates belonged to the branch of *N. oryzae* (Figure 3), which was consistent with the results obtained by using single-gene phylogenetic analysis. This reliably proved that the isolates were *N. oryzae*.

### 3.4. Screening of Antagonistic Bacteria

A total of 231 isolates of phyllosphere bacteria were isolated from the leaves of healthy *Chrysanthemum morifolium*. Twelve strains with antagonism were screened in total, of which nine strains were *Bacillus* (A10, D2, D5, D22, D31, D65, F70, F81 and F88), two strains were *Pseudomonas* (F5 and F17), and one strain was *Burkholderia* (D43). At the species level, the most common strain was *Bacillus amyloliquefaciens*, with a total of four strains (D5, D22, D31 and F88). The second was *Bacillus siamensis*, with three strains (D2, D65 and F81). The rest were: one strain of *Bacillus tequilensis* (A10), one strain of *Bacillus subtilis* (F70), one strain of *Pseudomonas citri*, and one strain of *Pseudomonas eucalypticola*. The test results of mannitol fermentation, glucose utilization under anaerobic conditions, amylolysis, nitrate reduction abilities, and V-P of each strain are shown in the Table 2. 

**Figure 4:**
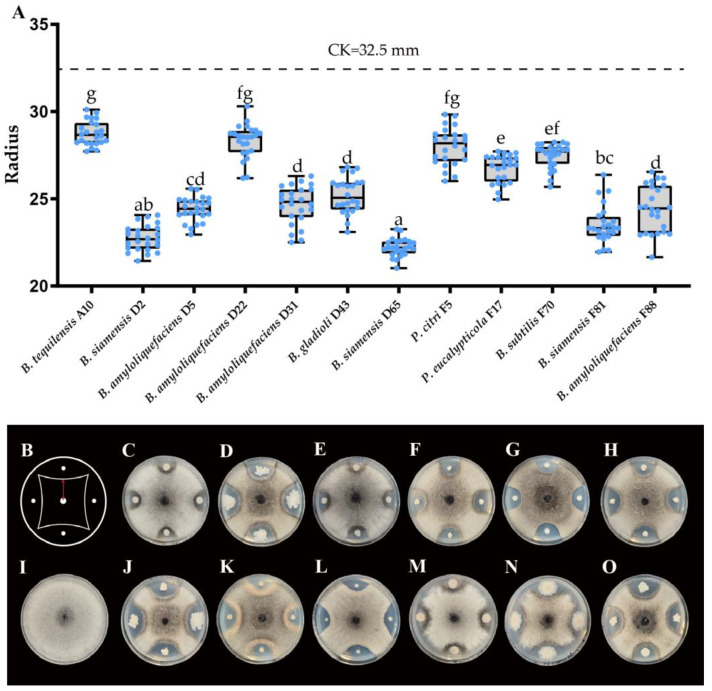
Inhibition effect of 12 strains on the growth of *N. oryzae*. (**A**) Colony radius of *N. oryzae* in dual culture assay on PDA plates treated by different strains. (**B**) Schematic diagram of dual culture assay, central position represents *N. oryzae*, and the four directions represent bacterial strains, the red line represents the colony radius of *N. oryzae*. (**C**) *Bacillus tequilensis* A10. (**D**) *Bacillus siamensis* D2. (**E**) *Bacillus amyloliquefaciens* D5. (**F**) *Bacillus amyloliquefaciens* D22. (**G**) *Bacillus amyloliquefaciens* D31. (**H**) *Burkholderia gladioli* D43. (**I**) Control. (**J**) *Bacillus siamensis* D65. (**K**) *Pseudomonas citri* F5. (**L**) *Pseudomonas eucalypticola* F17. (**M**) *Bacillus subtilis* F70. (**N**) *Bacillus siamensis* F81. (**O**) *Bacillus amyloliquefaciens* F88. Different letters above bars indicate a significant difference according to one-way ANOVA (*p* < 0.05).

At the dual culture assay on PDA plates, the plates treated with three *Bacillus siamensis* strains D2, D65, and D81, had the smallest fungal colony radius, indicating that *Bacillus siamensis* had the best antagonistic effect against *N. oryzae* among the 12 *C. morifolium* phyllosphere bacteria strains (Figure 4). The antagonistic effect of strain D65 was the best, with a radius of only 22.22 mm, which shows an extremely significant difference compared to the control (32.50 mm).

### 3.5. SPME-GC-Q-TOF Analysis of the D65 Fermentation Broth Components

**Figure 5:**
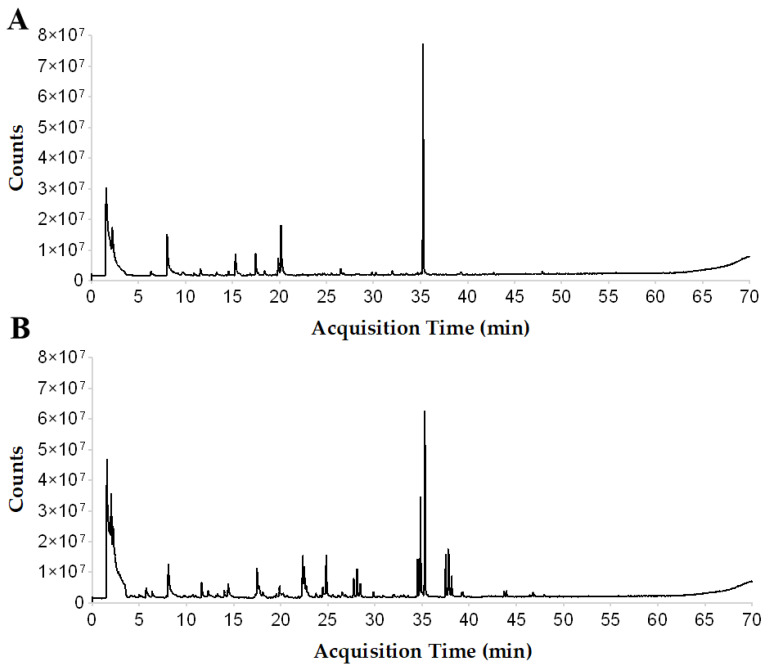
Chromatogram of SPME-GC-Q-TOF analysis. (**A**) LB medium. (**B**) *Bacillus siamensis* D65 fermentation broth.

To identify the components of the *Bacillus siamensis* D65 fermentation broth components which possessed the best antagonistic ability against *N. oryzae*, the SPME-GC-Q-TOF analysis of *Bacillus siamensis* D65 fermentation broth was carried out. It can be clearly seen that the chromatogram of the D65 fermentation broth has more numerous and complex peaks compared to LB medium. D65 has 218 compounds, while LB medium only has 110 compounds, indicating that D65 has abundant metabolic products (Figure 5). After deleting the repeated compounds and discarding compounds with a match factor below 90 and compounds containing silicon, there were still 38 compounds left. After removing the same compounds as were in LB medium, 22 specific compounds with a high match factor were obtained, including 2-Heptanone, 2-Nonanone, 3-Methyl-1-butanol and so on, which are listed in the table (Table 3). Although a large number of compounds were obtained in the results, further research is needed on the specific activity and function of each component.

## 4. Discussion

Plant diseases cause a considerable reduction in the yield of crops every year [33,34]. There are many pathogens that can infect chrysanthemums. In addition to chrysanthemum viruses and viroids, which often bring damage to chrysanthemums [35,36], white rust [37], anthracnose [38] and other leaf diseases also occur frequently. Chrysanthemums infected by pathogens, especially which cause leaf diseases, will lead to a decline not only the crop’s yield, but also its ornamental, edible and medicinal value, which will reduce its commercial characteristics and seriously hinder its commercial development [39].

The genus of *Nigrospora* is a widely distributed fungus, which can exist as an endophyte [40,41]. Many species can produce biologically active metabolites such as polyketides, terpenoids, steroids, N-containing compounds, and fatty acids [42]. Meanwhile, it also plays a role as a pathogen to affect plant health.

To date, a large number of studies have reported the diseases caused by the genus *Nigrospora*. From herbaceous plants such as *Oxalis corymbosa* to woody plants such as *Morus alba* [43,44], and from monocotyledonous plants such as *Triticum aestivum* to dicotyledonous plants such as *Nicotiana tabacum* [45,46], all can become the host of the genus *Nigrospora* (Supplementary Table S3). Moreover, not only can the fungus be hosted in the leaves but also in the stems and fruits of plants [47,48]. This demonstrates that the genus *Nigrospora* is an important pathogen with a wide host range and can be hosted in many parts of plants.

**Figure 6:**
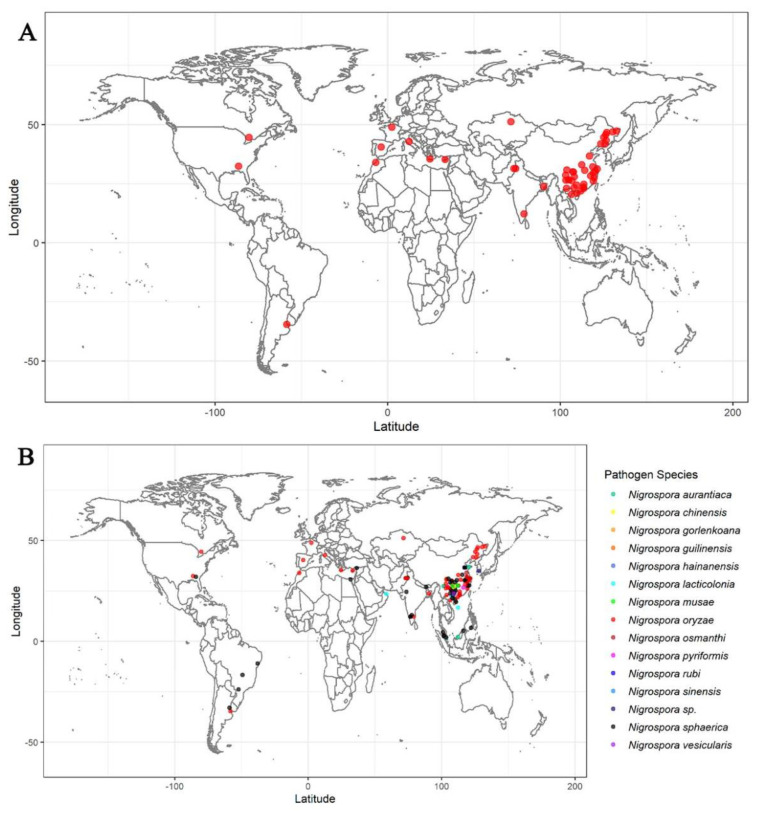
(**A**) Plant diseases caused by *N. oryzae* worldwide. (**B**) The occurrence of diseases caused by the genus *Nigrospora* worldwide.

On the world scale, the pathogens of the genus *Nigrospora* are widely distributed (Figure 6), and there are reports of plant diseases caused by the genus *Nigrospora* on all continents except for Oceania and Antarctica. The main distribution range of the disease caused by the genus *Nigrospora* is the tropical and temperate zones, and there are few relevant reports in the cold zone.

East and Southeast Asia, including China, have the highest numbers of reports, and the maximum diversity of species. This indicates that the genus *Nigrospora* may be a very important potential pathogen in China and its neighboring areas. In terms of the frequency and scope of occurrence, the most frequent and widespread species is *N. oryzae*, followed by *N. sphaerica*, while the other species mainly occur in East Asia. This fact implies that the research on *N. oryzae* and *N. sphaerica* is of great significance for the prevention and control of the diseases caused by the genus *Nigrospora*.

*N. oryzae* and *N. sphaerica* can often infect the same species of plant, such as *Arachis hypogaea* and *Vaccinium corymbosum* [49,50,51,52]. As for chrysanthemums, a widespread disease caused by *N. sphaerica* has been found in chrysanthemum growing areas [53], and in this study we found that *N. oryzae* is also a pathogen of chrysanthemum. This indicates that *N. oryzae* and *N. sphaerica* may have similar host ranges.

As a means of prevention and control of plant disease, biological control has been greatly promoted in today’s widespread use of chemical pesticides. Screening microorganisms with biocontrol effects from different parts is a good choice; for example, Wang et al. screened many antagonistic *Bacillus* strains from passion fruit leaves, which can prevent disease caused by *N. sphaerica* [29]. In this article, 12 biocontrol strains with antagonistic effects were screened from the leaves of *Chrysanthemum morifolium*, among which *Bacillus siamensis* D65 had the most significant antagonistic effect and could be further studied as an effective biocontrol resource.

Moreover, the metabolites were profiled using SPME-GC-Q-TOF. In total, 22 compounds were identified, and several of these compounds have been reported to be produced by bacteria or to possess antimicrobial activities and other functions. For example, 3-methyl-1-Butanol, produced by *Bacillus*, *Pseudomonas* or *Phoma*, can promote maize and wheat growth at appropriate concentrations [54]. Volatile compounds produced by *Tetrapisispora* sp. which contain Nonane, 2,6-dimethyl- have antifungal properties against *Botrytis cinerea* [55]. 2-Nonanol and 2-Nonanone can inhibit *Fusarium graminearum* growth and subspore germination, and prevent wheat scab caused by *Fusarium graminearum* [56,57]. 2-Undecanone has a targeted mode of antagonistic action against *Candida albicans* biofilm [58]. Fermentation broth produced by *Bacillus amyloliquefaciens* containing 2-Heptanone, 2-Nonanone, 2-Nonanol, 2-Undecanone, and 2-Undecanol has good antagonistic effects against plant pathogenic fungi such as *Monilinia fructigena*, *Glomerella cingulate*, *Rhizoctonia solani*, and so on [59].

SPME-GC-Q-TOF analysis in this study provided a variety of compounds that far exceeded the number of currently reported compounds which possess antagonistic effects. However, further research is needed to determine which type or types of compounds play a role in inhibiting plant fungal growth. The results of this study can be used as a reservoir to further screen components with antagonistic substances against pathogenic fungi.

## 5. Conclusions

In this study, *N. oryzae*, a pathogenic fungus with a wide host range, was identified as the pathogen of *C. morifolium* leaf spot disease. This is the first report of *C. morifolium* leaf disease caused by *N. oryzae* in the world. The result expanded our understanding of the host range that can be infected by *N. oryzae*. At the same time, this study screened 12 potential antagonistic strains with good antagonistic effects, which have inhibitory effects on *N. oryzae*, indicating the potential of these bacteria—especially *Bacillus siamensis* D65—as Biocontrol Agents (BCA) in controlling chrysanthemum diseases and other plants diseases caused by *N. oryzae*. Through SPME-GC-Q-TOF, 22 special compounds with antibacterial potential were identified, which can be studied as candidate compounds for inhibiting pathogenic fungi in a future study. Overall, this study provides a basis for further research and utilization of antagonistic strains for biological control.

## Figures and Tables

**Table 1:** Primers used in this study.

Loci	Primer	Sequence
ITS	ITS1 ITS4	CTTGGTCATTTAGAGGAAGTAA TCCTCCGCTTATTGATATGC
TUB2	Bt2a Bt2b	GGTAACCAAATCGGTGCTGCTTTC ACCCTCAGTGTAGTGACCCTTGGC
TEF1-α	EF1-728F EF2	CATCGAGAAGTTCGAGAAGG GGA(G/A)GTACCAGT(G/C)ATCATGTT
16s rRNA	27F 1492R	AGAGTTGATCCTGGCTCAG GTTACCTTGTTACGACTT

**Table 2:** Strains with antagonistic effects against *N. oryzae* screened from phyllosphere of *C. morifolium*. (Note: + represents positive, − represents negative).

Isolate	Hit Taxon	Hit Strain Name	Mannitol Fermentation	Glucose Utilization	Amylolysis	Nitrate Reduction	V-P
A10	*Bacillus tequilensis* (99.93%)	KCTC 13622	+	+	+	+	+
D2	*Bacillus siamensis* (99.45%)	KCTC 13613	+	+	+	+	+
D5	*Bacillus amyloliquefaciens* (99.93%)	NBRC 15535	−	+	+	+	+
D22	*Bacillus amyloliquefaciens* (99.93%)	MPA 1034	+	+	+	+	+
D31	*Bacillus amyloliquefaciens* (100%)	NBRC 15535	+	+	+	+	+
D43	*Burkholderia gladioli* (99.44%)	ATCC 10248	+	−	−	−	−
D65	*Bacillus siamensis* (99.59%)	KCTC 13613	−	+	+	+	+
F5	*Pseudomonas citri* (99.43%)	OPS 13-3	+	−	−	+	−
F17	*Pseudomonas eucalypticola* (99.93%)	CCTCC M2018494	−	−	−	−	−
F70	*Bacillus subtilis* (99.79%)	NBRC 13719	+	+	+	+	+
F81	*Bacillus siamensis* (99.86%)	KCTC 13613	+	+	+	+	+
F88	*Bacillus amyloliquefaciens* (100%)	NBRC 15535	−	+	+	+	+

**Table 3:** Components of *Bacillus siamensis* D65 fermentation broth (sorted by Component Area).

Component Area	Formula	Molecular Weight (g/mol)	Compound Name	CAS Number	Match Factor
41,649,590	C_13_H_28_O	200.36	2-Tridecanol	1653-31-2	94.94
33,930,144	C_4_H_8_O_2_	88.1	Ethyl Acetate	141-78-6	92.88
22,758,614	C_14_H_22_	190.32	Benzene, 1,3-bis(1,1-dimethylethyl)-	1014-60-4	97.49
20,428,923	C_11_H_24_O	172.31	2-Undecanol	1653-30-1	95.38
16,886,839	C_14_H_30_O	214.39	2-Tetradecanol	4706-81-4	91.53
15,412,517	C_13_H_26_O	198.34	2-Tridecanone	593-08-8	96.03
12,362,340	C_12_H_26_O	186.33	2-Dodecanol	10203-28-8	93.30
12,122,120	C_14_H_28_O	212.37	2-Tetradecanone	2345-27-9	93.44
8,805,195	C_12_H_24_O	184.32	2-Dodecanone	6175-49-1	94.47
8,767,459	C_9_H_20_O	144.25	2-Nonanol	628-99-9	90.61
6,409,912	C_7_H_14_O	114.19	2-Heptanone	110-43-0	95.02
5,336,527	C_11_H_22_O	170.29	2-Undecanone	112-12-9	94.43
4,158,934	C_9_H_18_O	142.24	2-Nonanone	821-55-6	92.25
2,147,246	C_15_H_30_O	226.4	2-Pentadecanone	2345-28-0	90.64
1,906,840	C_5_H_12_O	88.15	1-Butanol, 3-methyl-	123-51-3	92.62
1,713,221	C_11_H_24_	156.31	Nonane, 2,6-dimethyl-	17302-28-2	94.84
1,312,588	C_10_H_12_O	148.2	Benzaldehyde, 2,4,6-trimethyl-	487-68-3	90.80
1,272,686	C_11_H_18_N_2_	178.27	Pyrazine, 2,5-dimethyl-3-(3-methylbutyl)-	18433-98-2	92.33
1,073,632	C_13_H_28_	184.36	Undecane, 2,8-dimethyl-	17301-25-6	95.28
856,873	C_11_H_24_	156.31	Octane, 2,4,6-trimethyl-	62016-37-9	90.33
496,511	C_11_H_12_O_3_S	224.28	3-(Benzoylthio)-2-methylpropanoic acid	67714-34-5	92.59
242,368	C_23_H_44_O_3_	368.59	Carbonic acid, eicosyl vinyl ester	1000382-54-3	90.51

## Data Availability

Not applicable.

## Supplementary Materials

The following supporting information can be downloaded at: https://www.mdpi.com/article/10.3390/microorganisms11092224/s1, Table S1: GenBank accession numbers obtained in this study; Table S2: Reference sequences of *Nigrospora* species from GenBank used in phylogenetic analysis; Table S3: Hosts of the disease caused by genus *Nigrospora* and report sources.
