# Supplementary material for: Nigrospora oryzae Causing Leaf Spot Disease on Chrysanthemum × morifolium Ramat and Screening of Its Potential Antagonistic Bacteria
Source: Microorganisms. 2023 Sep 1;11(9):2224. doi: 10.3390/microorganisms11092224 (PMC10537370; doi:10.3390/microorganisms11092224)
Supplement: Supplementary file 1 [file microorganisms-11-02224-s001.zip › microorganisms-2558272-supplementary.pdf]

Supplementary Material

Table S1. GenBank accession numbers obtained in this study

No.	Strain	ITS	TUB2	TEF
1	JA	OQ860097	OQ921613	OQ921610
2	JE	OQ860098	OQ921614	OQ921611
3	JF	OQ860099	OQ921615	OQ921612

Table S2. Reference sequences of *Nigrospora* Species from GenBank used in phylogenetic analysis

No.	Species	Culture collection Number	ITS	TUB2	TEF1	Host Plant
1	*N. aurantiaca*	CGMCC 3.18130* = LC 7302	KX986064	KY019465	KY019295	*Nelumbo sp.*
2	*N. aurantiaca*	LC 7034	KX986093	KY019598	KY019394	*Musa paradisiaca*
3	*N. bambusae*	CGMCC 3.18327* = LC 7114	KY385307	KY385319	KY385313	*Bamboo*
4	*N. bambusae*	LC 7244	KY385306	KY385320	KY385314	*Bamboo*
5	*N. bambusae*	LC 7245	KY385305	KY385321	KY385315	*Bamboo*
6	*N. camelliae-sinensis*	LC 2710	KX985957	KY019484	KY019310	*Castanopsis sp.*
7	*N. camelliae-sinensis*	CGMCC 3.18125* = LC 3500	KX985986	KY019460	KY019293	*Camellia sinensis*
8	*N. camelliae-sinensis*	LC 4460	KX986015	KY019538	KY019353	*Castanopsis sp.*
9	*N. camelliae-sinensis*	LC 6304	KX986045	KY019566	KY019370	*Camellia sinensis*
10	*N. camelliae-sinensis*	LC 6984	KX986080	KY019587	KY019387	*Musa paradisiaca*
11	*N. camelliae-sinensis*	LC 6989	KX986083	KY019590	KY019453	*Musa paradisiaca*
12	*N. camelliae-sinensis*	LC12070	MN215773	MN329937	MN264012	*Saccharum officinarum*
13	*N. chinensis*	LC 2696	KX985947	KY019474	KY019424	*Lindera aggregata*
14	*N. chinensis*	LC 3085	KX985970	KY019497	KY019427	*Camellia sinensis*
15	*N. chinensis*	LC 4364	KX986011	KY019534	KY019435	*Aucuba japonica*

16	*N. chinensis*	LC 4433	KX986013	KY019536	KY019436	*Castanopsis sp.*
17	*N. chinensis*	LC 4565	KX986021	KY019544	KY019442	*Itea sp.*
18	*N. chinensis*	CGMCC 3.18127* = LC 4575	KX986023	KY019462	KY019422	*Machilus breviflora*
19	*N. chinensis*	LC 4619	KX986025	KY019547	KY019444	*Osmanthus sp.*
20	*N. chinensis*	LC 4660	KX986026	KY019548	KY019445	*Quercus sp.*
21	*N. chinensis*	LC 4673	KX986028	KY019550	KY019446	*Smilax ocreata*
22	*N. chinensis*	LC 6631	KX986043	KY019569	KY019448	*Camellia sinensis*
23	*N. chinensis*	LC 6972	KX986078	KY019585	KY019451	*Musa paradisiaca*
24	*N. falsivesicularis*	CGMCC 3.19678* = LC12067 T	MN215778	MN329942	MN264017	*Saccharum officinarum*
25	*N. falsivesicularis*	LC13553	MN215779	MN329943	MN264018	*Saccharum officinarum*
26	*N. gorlenkoana*	CBS 480.73*	KX986048	KY019456	KY019420	*Vitis vinifera*
27	*N. gorlenkoana*	JZB 3230001	MN495939	MN549381	MN544645	*Cirsium setosum*
28	*N. guilinensis*	CGMCC 3.18124* = LC 3481	KX985983	KY019459	KY019292	*Camellia sinensis*
29	*N. guilinensis*	LC 7301	KX986063	KY019608	KY019404	*Vitis vinifera*
30	*N. hainanensis*	CGMCC 3.18129* = LC 7030	KX986091	KY019464	KY019415	*Musa paradisiaca*
31	*N. hainanensis*	LC 6979	KX986079	KY019586	KY019416	*Musa paradisiaca*
32	*N. hainanensis*	LC13514	MN215780	MN329944	MN264019	*Saccharum officinarum*
33	*N. lacticolonia*	CGMCC 3.18123* = LC 3324	KX985978	KY019458	KY019291	*Camellia sinensis*
34	*N. lacticolonia*	LC 7009	KX986087	KY019594	KY019454	*Musa paradisiaca*
35	*N. lacticolonia*	LC12059	MN215783	MN329947	MN264022	*Saccharum officinarum*
36	*N. musae*	CBS 319.34*	KX986076	KY019455	KY019419	*Musa paradisiaca*
37	*N. musae*	LC 6385	KX986042	KY019567	KY019371	*Camellia sinensis*
38	*N. oryzae*	LC 6759	KX986054	KY019572	KY019374	*Oryza sativa*
39	*N. oryzae*	LC 6760	KX986055	KY019573	KY019375	*Oryza sativa*
40	*N. oryzae*	LC 7293	KX985931	KY019601	KY019396	*Nelumbo sp.*
41	*N. oryzae*	LC 7297	KX985936	KY019605	KY019400	*Nelumbo sp.*

42	*N. oryzae*	LC 2689	KX985942	KY019469	KY019423	*Rhododendron sp.*
43	*N. oryzae*	LC 2693	KX985944	KY019471	KY019299	*Neolitsea sp.*
44	*N. oryzae*	LC 2695	KX985946	KY019473	KY019301	*Rubus reflexus*
45	*N. oryzae*	LC 2699	KX985949	KY019476	KY019303	*Hamamelis mollis*
46	*N. oryzae*	LC 2707	KX985954	KY019481	KY019307	*Rhododendron simiarum*
47	*N. oryzae*	LC 2712	KX985958	KY019485	KY019311	*Castanopsis sp.*
48	*N. oryzae*	LC 2724	KX985959	KY019486	KY019312	*Symplocos zizyphoides*
49	*N. oryzae*	LC 2749	KX985962	KY019489	KY019315	*Ternstroemia sp.*
50	*N. oryzae*	LC 2752	KX985963	KY019490	KY019316	*Osmanthus sp.*
51	*N. oryzae*	LC 2972	KX985967	KY019494	KY019320	*Tutcheria microcarpa*
52	*N. oryzae*	LC 2991	KX985969	KY019496	KY019321	*Cleyera japonica*
53	*N. oryzae*	LC 4273	KX985995	KY019519	KY019336	*Cephalotaxus sinensis*
54	*N. oryzae*	LC 4294	KX986002	KY019526	KY019343	*Daphniphyllum macropodum*
55	*N. oryzae*	LC 4338	KX986008	KY019532	KY019349	*Camellia sp.*
56	*N. oryzae*	LC 4680	KX986030	KY019552	KY019357	*Camellia sinensis*
57	*N. oryzae*	LC 4961	KX986031	KY019553	KY019358	*Pittosporum illicioides*
58	*N. oryzae*	LC 5181	KX986032	KY019554	KY019359	*Pentactina rupicola*
59	*N. osmanthi*	CGMCC 3.18126* = LC 4350	KX986010	KY019461	KY019421	*Osmanthus sp.*
60	*N. osmanthi*	LC 4487	KX986017	KY019540	KY019438	*Hedera nepalensis*
61	*N. pyriformis*	CGMCC 3.18122* = LC 2045	KX985940	KY019457	KY019290	*Citrus sinensis*
62	*N. pyriformis*	LC 2688	KX985941	KY019468	KY019297	*Lindera aggregata*
63	*N. pyriformis*	LC 2690	KX985943	KY019470	KY019298	*Rosa sp.*
64	*N. pyriformis*	LC 2694	KX985945	KY019472	KY019300	*Rubus reflexus*
65	*N. pyriformis*	LC 3099	KX985971	KY019498	KY019322	*Camellia sinensis*
66	*N. pyriformis*	LC 4669	KX986027	KY019549	KY019355	*Castanopsis sp.*
67	*N. pyriformis*	LC 6985	KX986081	KY019588	KY019388	*Musa paradisiaca*

68	*N. pyriformis*	LC12075	MN215787	MN329988	MN264026	*Saccharum officinarum*
69	*N. rubi*	CGMCC 3.18326* = LC 2698	KX985948	KY019475	KY019302	*Rubus sp.*
70	*N. sphaerica*	LC 7294	KX985932	KY019602	KY019397	*Nelumbo sp.*
71	*N. sphaerica*	LC 2705	KX985952	KY019479	KY019305	*Rosa sp.*
72	*N. sphaerica*	LC 2839	KX985964	KY019491	KY019317	*Harpullia longipetala*
73	*N. sphaerica*	LC 2958	KX985966	KY019493	KY019319	*Cleyera japonica*
74	*N. sphaerica*	LC 3420	KX985980	KY019506	KY019325	*Camellia sinensis*
75	*N. sphaerica*	LC 4174	KX985989	KY019513	KY019330	*Rhododendron arboreum*
76	*N. sphaerica*	LC 4241	KX985990	KY019514	KY019331	*Deutzia sp.*
77	*N. sphaerica*	LC 6969	KX986077	KY019584	KY019386	*Musa paradisiaca*
78	*N. sphaerica*	PC KS4A1 C R2	MK408581	MK408565	MK408570	*Hylocereus polyrhizus*
79	*N. vesicularis*	CGMCC 3.18128* = LC 7010	KX986088	KY019463	KY019294	*Musa paradisiaca*
80	*N. vesicularis*	LC 0322	KX985939	KY019467	KY019296	*Unknown host plant*
81	N. vesicularifera	CGMCC 3.19333* = LC12052	MN215812	MN329975	MN264051	*Saccharum officinarum*
82	N. vesicularifera	LC13532	MN215813	MN329976	MN264052	*Saccharum officinarum*
83	*N. zimmermanii*	CBS 290.62*	KY385309	KY385317	KY385311	*Saccharum officinarum*
84	*N. zimmermanii*	CBS 984.69	KY385310	KY385322	KY385316	*Saccharum officinarum*
85	*N. zimmermanii*	LC12048	MN215818	MN329981	MN264057	*Saccharum officinarum*
Outgroup	*Arthrinium malaysianum*	CBS 102053	NR_120273	KF144988	KF145030	

Table S3. Hosts of the disease caused by genus *Nigrospora* and report places

编号	Species	Host	Location	Longitude	Latitude	Reference
1	*Nigrospora sphaerica*	*Chrysanthemum morifolium*	Tong xiang, Zhejiang Province, China	120.79	31.09	(Luo et al., 2022)
2	*Nigrospora lacticolonia*	*Hylocereus polyrhizus*	Port Dickson, Negeri Sembilan, Malaysia	101.8	2.52	(Kee et al., 2019)
3			Kluang, Johor, Malaysia	103.33	2.03	
4	*Nigrospora sphaerica*		Port Dickson, Negeri Sembilan, Malaysia	101.85	2.52	
5			Kluang, Johor, Malaysia	103.38	2.03	
6	*Nigrospora gorlenkoana*	Cirsium setosum	Shandong Peninsula, China	117	36.65	(Hao et al., 2020)
7	*Nigrospora oryzae*	*Phyllostachys nigra, Rudbeckia hirta, Scirpus sp.*	Shandong Peninsula, China	117.05	36.65	
8	*Nigrospora osmanthi*	*Rosa chinensis, Phragmites australis, Cirsium setosum, Phyllostachys nigra, Rudbeckia hirta*	Shandong Peninsula, China	117.1	36.65	
9	*Nigrospora rubi*	*Fraxinus sp.*	Shandong Peninsula, China	117.15	36.65	
10	*Nigrospora sphaerica*	*Cirsium setosum, Phragmites australis, Fraxinus sp.*	Shandong Peninsula, China	117.2	36.65	
11	*Nigrospora aurantiaca*	*Castanea mollissima*	Dechang, Sichuan Province, China	102.17	27.4	(Luo et al., 2020)
12			Yanyuan, Sichuan Province, China	101.51	27.42	

13	*Nigrospora sphaerica*	*Sesamum indicum*	Faisalabad, Punjab, Pakistan	73.07	31.43	(Rehman et al., 2022)
14	*Nigrospora sp.*	*Actinidia deliciosa*	Sacheon, South Korea	128	35	(Kwon et al., 2017)
15	*Nigrospora lacticolonia*	*Bougainvillea spectabilis*	Yongxing Island of Sansha City, Hainan Province, China	112.3	16.8	(Li et al., 2022)
16	*Nigrospora sphaerica*	*Phoenix dactylifera*	Basra Province, Philippines	121.97	6.7	(Abass et al., 2013)
17	*Nigrospora osmanthi*	*Ficus pandurata*	Suyang, Jiangsu Province, China	118.77	34.13	(Liu et al., 2019)
18	*Nigrospora guilinensis*	*Phellodendron chinense*	Dujiangyan, Sichuan Province, China	103.62	30.98	(Zeng et al., 2020)
19			Dayi, Sichuan Province, China	103.52	30.58	
20	*Nigrospora sphaerica*	*Phoenix dactylifera*	Lahore, Pakistan	74.36	31.55	(Alam et al., 2020)
21	*Nigrospora pyriformis*	*Chenopodium album*	Hezhang, Guizhou Province, China	104.64	27.08	(Chen et al., 2020)
22	*Nigrospora oryzae*	*Phaseolus vulgaris*	Longli, Guizhou Province, China	106.8	26.27	(Luo and Jiang, 2022)
23	*Nigrospora aurantiaca*	*Nicotiana tabacum*	Zhengan, Guizhou Province, China	107.43	28.55	(Huang et al., 2021)
24	*Nigrospora oryzae*	*Nelumbo nucifera*	Fuzho, Fujian Province, China	119.28	26.08	(Zhang et al., 2018)
25	*Nigrospora osmanthi*	*Fagopyrum tataricum*	Xiangxiang, Hunan Province, China	112.53	27.73	(Shen et al., 2021)
26	*Nigrospora oryzae*	*Photinia serrulata*	Guiyang, Guizhou Province, China	106.65	20.44	(He et al., 2019)
27	*Nigrospora pyriformis*	*Nelumbo nucifera*	Guangchang, Jiangxi Province, China	116.31	26.79	(Gong et al., 2022)

28	*Nigrospora oryzae*	*Arachis hypogaea*	Mekkalur, Thiruvannamalai, India	79.08	12.23	(Vijayalakshmi et al., 2022)
29	*Nigrospora osmanthi*	*Stenotaphrum secundatum*	Haikou, Hainan Province, China	110.32	20.05	(Mei et al., 2019)
30	*Nigrospora sphaerica*	*Cyclocarya paliurus*	Anhui Province, China	117.29	30.24	(Zheng et al., 2021)
31			Guangxi Province, China	109.92	26.02	
32			Hubei Province, China	111.33	30.43	
33			Nanjing, Jiangsu Province, China	118.82	32.08	
34	*Nigrospora sphaerica*	*Arachis hypogaea*	Wenchang, Hainan Province, China	110.76	19.62	(Liu et al., 2020)
35	*Nigrospora hainanensis*	*Oxalis corymbosa*	Nanning, Guangxi Province, China	108.32	22.82	(Zheng et al., 2022)
36	*Nigrospora sphaerica*	*Glycyrrhiza glabra*	Udaipur, India	73.68	24.58	(Verma and Gupta, 2008)
37	*Nigrospora sphaerica*	*Camellia oleifera*	Shexian, Anhui Province, China	118.42	29.86	(Liu et al., 2020)
38	*Nigrospora sphaerica*	*Saccharum spp.*	Guangzhou, Guangdong Province, China	113.23	23.16	(Cui et al., 2018)
39	*Nigrospora oryzae*	*Zingiber officinale*	Nanning, Guangxi Province, China	108.07	23.24	(Liu et al., 2022)
40	*Nigrospora oryzae*	*Calibrachoa hybrida*	Hurlingham, Argentina	-58.4	-34.6	(Borrelli et al., 2020)
41	*Nigrospora oryzae*	*Centranthera cochinchinensis*	Pingbian, Yunnan Province, China	103.76	23.05	(Zhai et al., 2019)
42	*Nigrospora oryzae*	*Vaccinium corymbosum*	Shanghai City, China	121.47	31.23	(Zhang et al., 2019)
43	*Nigrospora sphaerica*	*Morus alba*	Mysuru, India	76.63	12.26	(Arunakumar et al., 2019)
44	*Nigrospora oryzae*	*Commelina communis*	Dianjiang, Chongqing City, China	107.3	30.06	(Qiu et al., 2022)

45	*Nigrospora osmanthi*	*Orthosiphon stamineus*	Serdang, Selangor, Malaysia	101.72	3	(Ismail et al., 2022)
46	*Nigrospora oryzae*	*Triticum aestivum*	Astana Province, Kazakhstan	71.47	51.16	(Eken et al., 2016)
47	*Nigrospora oryzae*	*Bassia scoparia*	Punjab Province, Pakistan	74.21	31.33	(Anjum et al., 2021)
48	*Nigrospora lacticolonia*	*Phoenix dactylifera*	Oman	58.62	23.6	(Al-Nadabi et al., 2020)
49	*Nigrospora sphaerica*	*Adenium obesum*	Goiânia, Goiás state, Brazil	-49.29	-16.67	(de Jesus et al., 2020)
50	*Nigrospora sphaerica*	*Passiflora edulis*	Huishui, Guizhou Province, China	106.83	106.5	(Wang et al., 2022)
51	*Nigrospora oryzae*	*Oryza rufipogon*	Zhanjinag, Guangdong Province, China	109.79	20.93	(Liu et al., 2021)
52	*Nigrospora sphaerica*	*Selenicereus megalanthus*	Sabah, Malaysia	116.29	5.35	(Khoo et al., 2022)
53	*Nigrospora sphaerica*	*Heliocarpus americanus*	Beltrão, Paraná, Brazil	-52.27	-23.8	(Bernardi et al., 2021)
54	*Nigrospora oryzae*	*Populus alba L. × P. berolinensis Dipp.*	Shenyang, Liaoning Province, China	123.57	41.82	(Zhang et al., 2022)
55	*Nigrospora oryzae*	*Davidia involucrata*	Meigu, Sichuan Province, China	103.23	28.55	(Yang et al., 2022)
56	*Nigrospora sphaerica*	*Platycodon grandiflorus*	Anhui Province, China	115.42	33.13	(Chen et al., 2022)
57	*Nigrospora chinensis*	*Camellia oleifera*	Tianzhu, Guizhou Province, China	109.21	26.91	(Qin et al., 2021)
58	*Nigrospora sphaerica*	*Citrullus lanatus*	Serdang, Selangor, Malaysia	101.77	3	(Ismail and Abd Razak, 2021)
59	*Nigrospora oryzae*	*Pennisetum purpureum*	Fengdu, Chongqing City, China	107.73	29.86	(Han et al., 2020)

60	*Nigrospora sphaerica*	*Eclipta prostrata*	Dianjiang, Chongqing City, China	107.35	30.06	(Qiu et al., 2022)
61	*Nigrospora sphaerica*	*Vigna unguiculata*	Karnataka, India	77.57	12.97	(Deepika et al., 2021)
62	*Nigrospora sphaerica*	*Cunninghamia lanceolata*	Qingyang County, Anhui Province, China	117.84	36.64	(Xu et al., 2017)
63	*Nigrospora oryzae*	*Gossypium hirsutum*	Alabama, America	-86.33	32.37	(Palmateer et al., 2003)
64	*Nigrospora oryzae*	*Nicotiana tabacum*	Tongren, Guizhou province, China	108.12	27.99	(Wang et al., 2022)
65	*Nigrospora aurantiaca*	*Saccharum offificinarum*	Chongzuo, Guangxi Province, China	107.36	22.37	(Raza et al., 2019)
66			Guilin, Guangxi Province, China	110.3	25.27	
67			Laibin, Guangxi Province, China	109.23	23.73	
68	*Nigrospora sinensis*		Chongzuo, Guangxi Province, China	107.41	22.37	
69			Nanning, Guangxi Province, China	108.37	22.82	
70			Guangzhou, Guangdong Province, China	113.23	23.16	
71	*Nigrospora gorlenkoana*		Liuzhou, Guangxi Province, China	109.42	24.33	
72	*Nigrospora lacticolonia*		Chongzuo, Guangxi Province, China	107.31	22.37	
73			Nanning, Guangxi Province, China	108.42	22.84	

74			Guangzhou, Guangdong province, China	113.18	23.18	
75			Zhanjiang, Guangdong Province, China	109.81	20.95	
76	*Nigrospora oryzae*		Liuzhou, Guangxi Province, China	109.44	24.35	
77	*Nigrospora sphaerica*		Baise, Guangxi Province, China	106.62	23.89	
78			Fangchenggang, Guangxi Province, China	108.2	21.37	
79			Guilin, Guangxi Province, China	110.35	25.29	
80			Hechi, Guangxi Province, China	108.06	24.69	
81			Laibin, Guangxi Province, China	109.28	23.75	
82			Liuzhou, Guangxi Province, China	109.49	24.35	
83			Nanning, Guangxi Province, China	108.27	22.84	
84			Guangzhou, Guangdong Province, China	113.33	23.2	
85	*Nigrospora vesicularis*		Laibin, Guangxi Province, China	109.18	23.73	
86	*Nigrospora sphaerica*	*Mentha canadensis*	Chengmai, Hainan Province, China	119.29	29.48	(Sun et al., 2020)
87	*Nigrospora oryzae*	*Hibiscus mutabilis*	Chengdu, Sichuan Province, China	103.86	30.71	(Han et al., 2021)
88	*Nigrospora sphaerica*	*Vaccinium corymbosum*	Buenos Aires Province, Argentina	-59	-33	(Wright et al., 2008)
89			Entre Ríos Province, Argentina	-38	-11	

90	*Nigrospora sphaerica*	*Akebia trifoliata*	Zhangjiajie, Hunan Province, China	110.2	29.4	(Hong et al., 2021)
91	*Nigrospora sphaerica*	*Parthenium hysterophorus*	Perak, Malaysia	101.09	4.59	(Zafri et al., 2021)
92	*Nigrospora oryzae*	*Costus speciosus*	Chengmai, Hainan Province, China	119.29	29.48	(Sun et al., 2021)
93	*Nigrospora oryzae*	*Poa pratensis*	Ontario, Canada	-80.25	44.55	(Zheng et al., 2012)
94	*Nigrospora sphaerica*	*Morus alba*	Santai, Sichuan Province, China	105.08	31.1	(Chen et al., 2018)
95	*Nigrospora oryzae*	*Aloe vera*	Dhaka, Bangladesh	90.41	23.81	(Begum et al., 2018)
96	*Nigrospora sphaerica*	*Lagenaria siceraria*	Sumter County, Georgia	-84.23	32.07	(Li et al., 2016)
97	*Nigrospora oryzae*	*Arundo donax*	France	2.42	48.87	(Widmer et al., 2006)
98			Crete	24.53	35.42	
99			Cyprus	33.38	35.17	
100			Italy	12.5	42.8	
101			Morocco	-6.84	34.02	
102			Spain	-3.75	40.42	
103	*Nigrospora oryzae*	*Aloe vera*	Faisalabad , Punjab Province, Pakistan	73.09	31.42	(Alam et al., 2017)
104	*Nigrospora oryzae*	*Actinidia deliciosa*	Taishun, Zhejiang Province, China	119.72	27.57	(Li et al., 2018)
105			Liupanshui, Guizhou Province, China	104.83	26.59	
106	*Nigrospora sphaerica*	*Camellia sinensis*	Darjeeling, India	88.26	27.04	(Dutta et al., 2015)

107	*Nigrospora oryzae*	*Euonymus japonicas*	Nanyang, Henan Province, China	112.55	33	(Xu et al., 2022)
108	*Nigrospora sphaerica*	*Hylocereus undatus*	Guangdong Province, China	110.01	21.01	(Liu et al., 2016)
109	*Nigrospora oryzae*	*Dendrobium candidum*	Hangzhou, Zhejiang Province, China	120.19	30.26	(Wu et al., 2014)
110	*Nigrospora sphaerica*	*Actinidia sp.*	Taishun, Zhejiang Province, China	119.77	27.57	(Li et al., 2018)
111			Liupanshui, Guizhou Province, China	104.88	26.59	
112	*Nigrospora sphaerica*	*Wisteria sinensis*	Hatay, Turkey	36.25	36.42	(Soylu et al., 2011)
113	*Nigrospora sphaerica*	*Curcuma longa*	Ruian, Zhejiang Province, China	120.65	27.78	(Zhang et al., 2011)
114	*Nigrospora sphaerica*	*Camellia sinensis*	Qingyang, Anhui Province, China	117.89	30.65	(Liu et al., 2016)
115	*Nigrospora oryzae*	*Aloe vera*	Wuhan, Hubei Province, China	114.29	30.58	(Zhai et al., 2013)
116	*Nigrospora oryzae*	*Gossypium hirsutum*	Qianshan, Anhui Province, China	117.7	28.32	(Zhang et al., 2012)
117	*Nigrospora aurantiaca*	*Pandanus amaryllifolius*	Luyang, Sabah, Malaysia	112.3	2.3	(Khoo et al., 2022)
118	*Nigrospora sphaerica*	*Cenchrus purpureus Schumach., syn. Pennisetum purpureum Schumach*	Chongqing City, China	106.33	29.35	(Han et al., 2019)
119	*Nigrospora oryzae*	*Oryza sativa*	Jilin, Jilin Province, China	126.67	43.33	(Sun and Bai, 2018)
120			Dehui, Jinlin Province, China	125.23	44.53	
121			Tonghua, Jilin Province, China	125.94	41.73	

122	*Nigrospora oryzae*	*Oryza sativa*	Harbin, Heilongjiang Province, China	126.52	45.8	(Zhao et al., 2022)
123			kiamusze, Heilongjiang Province, China	130.34	46.82	
124			Suihua, Heilongjiang Province, China	126.99	46.66	
125			Jiansanjiang, Heilongjiang Province, China	132.63	47.26	
126	*Nigrospora oryzae*	*Oryza sativa*	Yangjiang, Guangdong Province, China	111.98	21.86	(Feng et al., 2013)
127			Shaoguan, Guangdong Province, China	113.61	24.68	
128			Dongguan, Guangdong Province, China	113.75	23.02	
129			Guangzhou, Guangdong Province, China	113.59	23.55	
130	*Nigrospora oryzae*	*Fagopyrum esculentum*	Huize, Yunnan Province, China	103.3	26.42	(Lu et al., 2022)
131	*Nigrospora sphaerica*	*Mangifera indica*	El Sharkia governorate, Egypt	31.8	30.8	(Youssef et al., 2022)
132	*Nigrospora musae*	*Basella alba*	Shuangfeng, Hunan Province, China	111.95	27.69	(Liao et al., 2023)
133	*Nigrospora oryzae*	*Dioscorea spp.*	Nanjing, Jiangsu Province, China	118.87	32.08	(Lu et al., 2023)
134	*Nigrospora sphaerica*	*Crossandra infundibuliformis*	Mysore, India	76.65	12.3	(Tejaswini et al., 2022)
135	*Nigrospora aurantiaca*	*Arachis hypogaea*	Laixi, Shandong Province, China	120.52	36.89	(He et al., 2022)

136	*Nigrospora musae*	*Zinnia elegans*	Shibing, Guizhou Province, China	108.2	27.52	(Liu et al., 2022)

Table 3 was used to draw a map of the diseases caused by the genus *Nigrospora* reported worldwide in recent years. The references were collected from Web of Science and CNKI. The coordinates of the disease occurrence place were from the reference or obtained from search in Internet according the occurrence location in references. When multiple data appear at the same coordinate point, the coordinate value were fine tuned to avoid overlap points on the map. The map in this paper was draw using ggplot2, ggmap, maps and maptools libraries of R (version 4.1.2).
